# Is La_3_Ni_2_O_6.5_ a Bulk Superconducting Nickelate?

**DOI:** 10.1021/acsami.3c17376

**Published:** 2024-02-21

**Authors:** Ran Gao, Lun Jin, Shuyuan Huyan, Danrui Ni, Haozhe Wang, Xianghan Xu, Sergey L. Bud’ko, Paul Canfield, Weiwei Xie, Robert J. Cava

**Affiliations:** † Department of Chemistry, Princeton University, Princeton, New Jersey 08544, United States; ‡ Ames National Laboratory, Iowa State University, Ames, Iowa 50011, United States; § Department of Physics and Astronomy, Iowa State University, Ames, Iowa 50011, United States; ∥ Department of Chemistry, Michigan State University, East Lansing, Michigan 48824, United States

**Keywords:** High pressure, nickelates, superconductivity

## Abstract

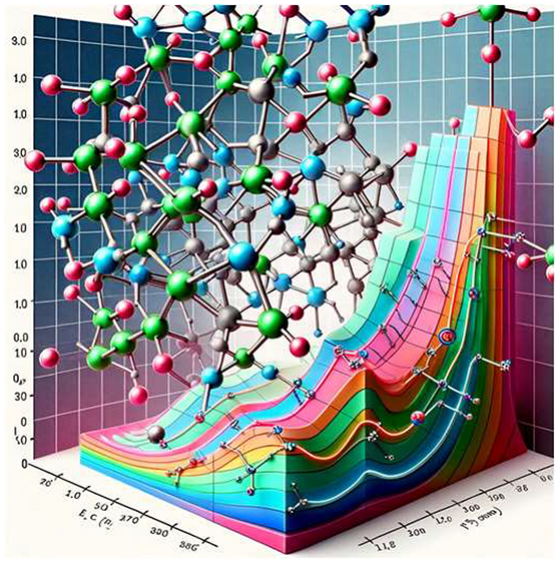

Superconducting states onsetting at moderately high temperatures have been observed in epitaxially stabilized *RE*NiO_2_-based thin films. However, recently, it has also been reported that superconductivity at high temperatures is observed in bulk La_3_Ni_2_O_7-δ_ at high pressure, opening further possibilities for study. Here we report the reduction profile of La_3_Ni_2_O_7_ in a stream of 5% H_2_/Ar gas and the isolation of the metastable intermediate phase La_3_Ni_2_O_6.45_, which is based on Ni^2+^. Although this reduced phase does not superconduct at ambient or high pressures, it offers insights into the Ni-327 system and encourages future study of nickelates as a function of oxygen content.

## Introduction

Since the discovery of superconducting cuprates late in the last century,^1^ nickelates have been predicted to potentially host this exotic state of matter,^2^ primarily because Ni, a magnetic element, is one of the closest neighbors of Cu on the periodic table. Despite much experimental effort on nickelates,^3,4^ superconductivity in epitaxially stabilized doped *RE*NiO_2_ thin films (topochemically reduced from the parent 113 perovskites) has only recently been discovered.^5−8^ This superconductivity has not been seen in bulk materials. Very recently, however, superconductivity near 80 K has been reported for La_3_Ni_2_O_7-δ_ in bulk form at high pressures.^9−12^ This advancement, if true, not only extends superconducting nickelates to bulk materials with a much higher critical temperature but also paves the way toward more possible candidate materials that should be examined. While some may argue that we have thus entered the “nickelate superconductor age”, there are still many challenges ahead that can be taken as a certainty.

In the present study, we characterize the reduction of the bilayer nickelate La_3_Ni_2_O_7_, using thermogravimetric analysis. Its reduction in forming gas (in our case 5% H_2_ in Ar) while maintaining its basic crystal structure^13−15^ is highly unusual for a transition metal oxide, which is due to the stability of Ni^2+^ in oxides, yielding a better than average possibility for changing its carrier concentration. In addition to the equilibrium behavior, we report an unusual kinetic effect on the phase assemblage observed on reduction. Finally, we have successfully isolated the metastable intermediate phase La_3_Ni_2_O_6.45_, a composition that is a member of the La_3_Ni_2_O_7-δ_ family, and have investigated its structural and physical properties. By removing about 0.5 oxygen per formula unit from the parent compound, we have introduced an intrinsic magnetic transition instead of superconductivity. We have looked for signs of superconductivity in this reduced phase at both ambient and high pressures but have not seen any. Even though the superconductivity is absent in our material, our results indicate that the investigation of difficult-to-reduce oxides should be of future interest.

## Experimental Section

Polycrystalline samples of La_3_Ni_2_O_7_ were synthesized by a high-temperature ceramic method using La_2_O_3_ (Alfa Aesar, 99.99%) and NiO (Sigma-Aldrich, ∼325 mesh, 99% with 1% unspecified impurity) as the starting materials. La_2_O_3_ was predried overnight in a furnace at 900 °C. Stoichiometric amounts (in metals) of the starting materials were weighed accurately and ground together thoroughly before being transferred into an alumina crucible. The mixed starting materials were reacted in a furnace at 1100 °C for 5 days in air with intermittent grinding. Both heating and cooling were set at a rate of 3 °C per minute. Reduced samples with a series of formulas of the type La_3_Ni_2_O_7–*x*_ were attained by the thermogravimetric analysis (TGA) technique with a TA Instruments TGA 5500. The parent La_3_Ni_2_O_7_ phase was reduced in a forming gas of 5% H_2_ in Ar at different temperatures ranging from 300 to 800 °C and held isothermally for various periods of time. A ramp rate of 10 °C per minute was used during heating, and fast cooling to ambient temperature was used after the isothermal segment.

Powder X-ray diffraction (PXRD) measurements were performed by using a Bruker D8 FOCUS diffractometer (Cu Kα radiation) at ambient temperature. PXRD data were collected after each intermittent grinding and TGA reduction to monitor the reaction process. Once the pure phase was attained, the PXRD data were collected at room temperature over a 2θ range from 5 to 110°, with much better statistical significance, for the following Le Bail refinements conducted by using TOPAS software.

The physical properties of all polycrystalline samples were analyzed by a Quantum Design Dynacool Physical Property Measurement System (PPMS). Magnetic measurements were performed by the PPMS, which was equipped with a vibrating sample magnetometer (VSM). Magnetic susceptibility (χ) is defined as *M*/*H*, where *M* is magnetization and *H* the applied magnetic field intensity. Heat capacity was measured by a standard relaxation method over a temperature range from 2 to 70 K under 0 T field.

High pressure electrical resistivity measurements using the Van der Pauw method were performed in a commercial Diamond Anvil cell (DAC)^16^ that fits the Quantum Design Physical Property Measurement System (PPMS). Standard cut-type Ia diamonds with a culet size of 400 μm were utilized as the anvils, with a 250 μm thick stainless-steel aperture disc served as the gasket. To ensure electrical insulation, the aperture was filled and compressed with a mixture of cubic boron nitride (cBN) and epoxy, and the rest of the gasket’s top surface was covered by STYCAST. Subsequently, a central hole with a diameter of 150 μm was drilled through the cBN layer to create a sample chamber. This chamber was initially loaded with a fine powder of NaCl, which acted as the pressure-transmitting medium (PTM). The NaCl powder was then compressed from ∼1 to 2 GPa until the hole became totally transparent. After that, an additional approximately 80 μm half-drilled hole was created within the NaCl layer. The La_3_Ni_2_O_7-δ_ powder sample was carefully introduced and pressed (2.1 GPa) into the half-drilled hole, with a small ruby sphere placed at the bottom of the sample as the manometer. Pressure was determined by the R_1_ line position of the ruby fluorescent spectra.^17^ Platinum foil electrodes were employed to establish electrical connections with the sample.

## Results and Discussion

**Figure 1:**
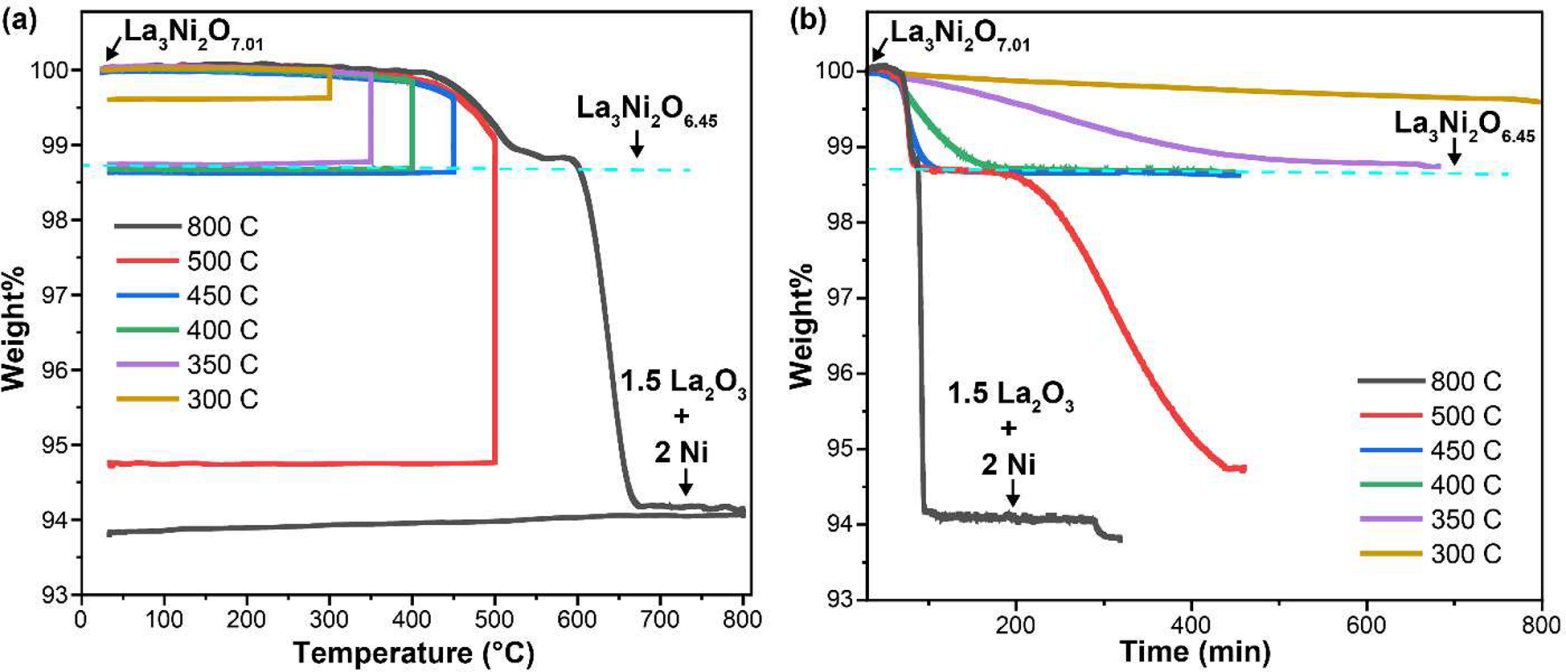
TGA reduction profile of La_3_Ni_2_O_**7**_. The La_3_Ni_2_O_7_ samples were reduced in forming gas (5% H_2_/Ar) under different reaction conditions. The resulting sample weight percents (obtained in the TGA) are plotted against (a) temperature and (b) time. (The time and temperature curves are from the same experiment.)

Reduced phases of La_3_Ni_2_O_7_ were prepared by using different TGA sequences, with the weight % variations plotted against both the temperature and time in Figure 1a and b. In general, the as-made La_3_Ni_2_O_7_ phase was reduced in a forming gas of 5% H_2_ in Ar during the isothermal segment that lasts for 3–10 h, followed by a fast-cooling process. As shown in Figure 1, the as-made La_3_Ni_2_O_7_ phase started to lose mass continuously at 300 °C and did not reach a final, stable oxygen content even after a 10 h isothermal reduction period. Therefore, it was necessary to increase the reduction temperature. A long-time stable weight occurred when the dwelling temperature was set to 350, 400, and 450 °C, respectively. Although a dwelling period of 10 h was required for the 350 °C sequence to reach a relatively constant mass, the samples reduced at 400 and 450 °C reached a stable mass within a 4–6 h isothermal reduction period. When the reduction temperature was elevated to 500 °C, the La_3_Ni_2_O_7_ sample reached the same stable mass first, and after staying at this plateau for approximately 2 h, a further mass loss began. Thus, at 500 C, the stable mass material is attainable, but there is a clear kinetic effect at play. The weight loss curve did not reach a new stable value after the 6 h isothermal reduction at 500 °C. For full reduction and decomposition to La_2_O_3_ plus elemental Ni, the La_3_Ni_2_O_7_ sample was reduced at 800 °C, and the weight change curve reached a new platform with a 3 h isothermal step, corresponding to an approximately 6% loss of the total weight. Although the “intermediate mass platform” can still be vaguely spotted in the 800 °C curve, it is obviously shorter compared to that present for the lower temperature reductions. From the weight % versus time plot (Figure 1b), we can observe that higher temperatures speed up the reduction process. In addition to the reduction temperature, the annealing time also contributes to the reduction process of La_3_Ni_2_O_7_, which is most obvious from in the 500 °C TGA run, as the “intermediate platform mass” only lasted for 2 h, while further annealing at this temperature gradually destroys this phase, making it metastable. By using PXRD to examine the sample residue after each TGA run, we found that the structural lattice of La_3_Ni_2_O_7_ had collapsed and reduced to the nontopochemical decomposition products La_2_O_3_ and Ni at 800 °C; hence, the formula of the as-made sample, La_3_Ni_2_O_7.01_, can be calculated precisely based on the weight loss from the 800 °C TGA curve. Therefore, the oxygen stoichiometry of the intermediate topochemically reduced phase is determined as La_3_Ni_2_O_6.45_ based on the weight % loss at the first plateau, which can be clearly observed in the TGA curves obtained at 400 and 450 °C (Figure 1a). This composition we take as being within the error of La_3_Ni_2_O_6.5_, which may be metastable for this material. At this composition the Ni would all be present in the 2+ state, a highly stable *d*^8^ configuration for the ionized nickel.^18,19^

**Figure 2:**
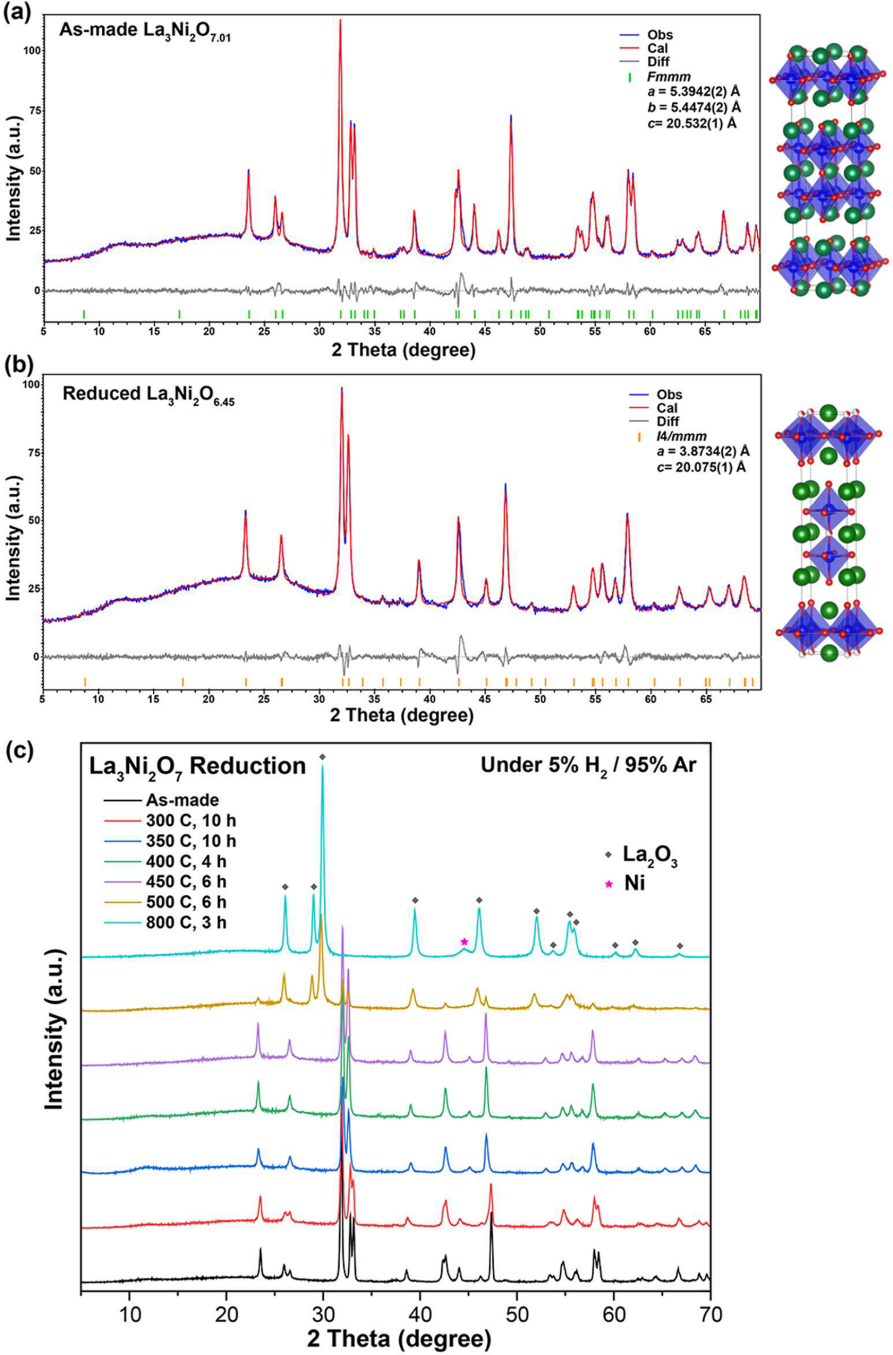
Structural analysis. The Le Bail fitting against the PXRD pattern collected from (a) the as-made La_3_Ni_2_O_7.01_ phase and (b) the reduced La_3_Ni_2_O_6.45_ phase. (c) PXRD patterns of the post-TGA samples made under different reduction conditions.

PXRD patterns of the postreduction powder samples were collected to analyze the structural change, and Le Bail fittings were conducted on the as-made and reduced phases to determine the symmetry and size of the unit cells. The as-made parent La_3_Ni_2_O_7.01_ phase can be indexed by a face-centered orthorhombic unit cell (space group *Fmmm*) with lattice parameters *a* = 5.3924(2) Å, *b* = 5.4474(2) Å, and *c* = 20.532(1) Å (Figure 2a), in good agreement with previous reports.^13^ The body-centered tetragonal unit cell (space group *I*4/*mmm*) that has been widely adopted for reduced phases of La_3_Ni_2_O_7-δ_ in the literature^14,15^ can be used to index our metastable reduced phase La_3_Ni_2_O_6.45_ as well, with lattice parameters *a* = 3.8734(2) Å and *c* = 20.075(1) Å (Figure 2b). This transition in symmetry from orthorhombic to tetragonal, caused by the oxygen deficiency, is consistent with previous reports.^13,14^ It is noted that after the √2 expansion of the tetragonal unit cell of the reduced phase, its parameter *a* (5.48 Å) is only marginally enlarged compared to the parameters *a* and *b* of the orthorhombic unit cell of the parent compound, while parameter *c* shrinks by approximately 0.5 Å upon the reduction. Based on the published structural refinements,^13,14^ the oxygen vacancies in the reduced phases are not randomly distributed. In general, they prefer to sit in the bridging apical oxygen site that connects the two Ni centers along the *c*-axis, in the La–O planes (The 4*a* site in the *Fmmm* unit cell and the 2*a* site in the *I*4/*mmm* unit cell). This can also explain the larger change in lattice parameter *c* on reduction.

Using the results of the PXRD data collected from the post-TGA samples reduced at different temperatures, more detailed insight into the reduction behavior of La_3_Ni_2_O_7_ can be revealed. As shown in Figure 2c, the reduction started at 300 °C in the 5% H_2_/Ar forming gas, though La_3_Ni_2_O_7_ did not fully convert to the tetragonal reduced phase after a 10 h isothermal process. The incomplete structural transition can be diagnosed by the partial merging of peaks (such as the doublets at 27°, 33°, 43°, and 58°) and the asymmetric peak shape (near 47°) when comparing the black and red patterns in Figure 2c, which also agrees well with the TGA results. The highly similar PXRD patterns of the post-TGA samples between 350 and 450 °C align well with the constantly observed metastable reduced La_3_Ni_2_O_7-δ_ phase in those TGA runs, indicating that the samples reduced at those temperatures have the same composition, La_3_Ni_2_O_6.45_. In the 500 °C pattern, the peaks representing La_2_O_3_ and Ni start to show up, while the crystallinity of the target La_3_Ni_2_O_6.45_ phase is highly compromised. This observation is mutually supported by the second weight % drop after the 2 h plateau in the 500 °C TGA curve, confirming that the structural lattice disintegrates upon prolonged heating at this temperature. The peaks of the La_3_Ni_2_O_6.45_ phase completely disappear in the 800 °C pattern; the pattern is now composed of only La_2_O_3_ and Ni peaks, indicating a thorough decomposition of the ternary compound. The continuously evolving peaks in the PXRD patterns from the as-made sample to the 450 °C reduced one reveal that the structural transition occurs through a series of compositions with a short-range distribution of different oxygen contents, which is clearly distinguishable from the final nontopochemical decomposition of the structural lattice (patterns 500 to 800 °C).

**Figure 3:**
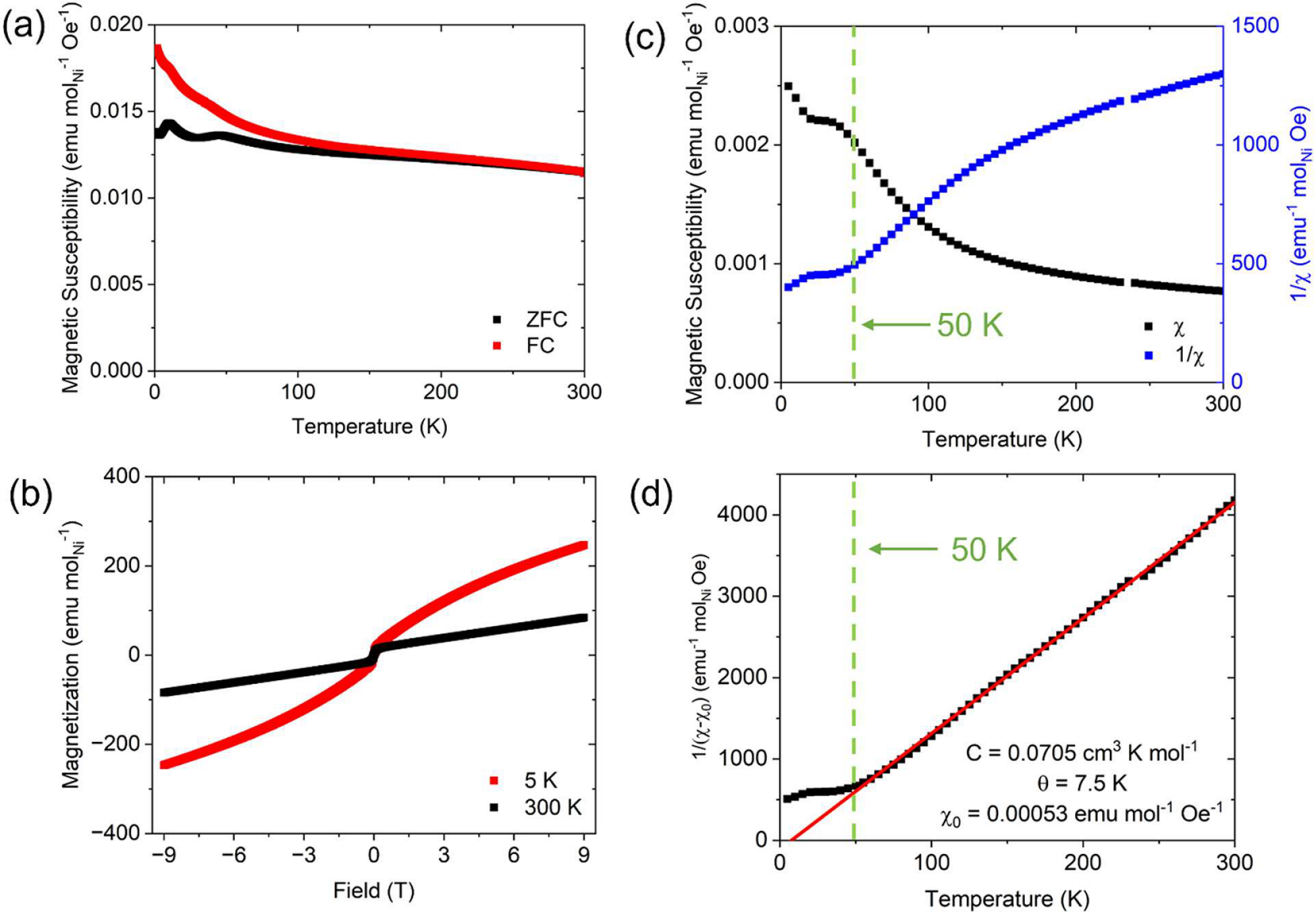
Magnetization of La_3_Ni_2_O_6.45_. (a) Magnetic susceptibility derived from the temperature-dependent magnetization data plotted against temperature (*H* = 1000 Oe). (b) Field-dependent magnetization data collected at 5 and 300 K. Note that the 300 K data allows us to estimate that the amount ferromagnetic impurity (if elemental Ni is in the reduced material, it is a very small amount, 0.43% per mole of La_1.5_NiO_3.225_). (c) Ferro-subtracted magnetic susceptibility (black) and the inverse of magnetic susceptibility (blue). (d) Curie–Weiss fit to the high-temperature region of the χ_0_ corrected 1/χ vs temperature plot from panel c.

Temperature-dependent magnetization data (*M*) were collected from the reduced phase La_3_Ni_2_O_6.45_, synthesized at 400 °C, using both zero-field-cooled (ZFC) and field-cooled (FC) approaches using an applied field of *H* = 1000 Oe. To avoid any potential ambiguities, the collected magnetization data were all scaled to per-mole-Ni (based on the formula La_1.5_NiO_3.225_). The resulting magnetic susceptibility (*M*/*H*) data are plotted against the temperature in Figure 3a. The ZFC and FC curves diverge from each other in the high-temperature regime. The ZFC curve distinctively shows two transitions—at temperatures of about 10 and 50 K, with the latter being much broader than the former. The field-dependent data were collected from the reduced phase at both 5 and 300 K over the field range −9 T ≤ *H* ≤ 9 T. The 300 K magnetization isotherm exhibits a “kink” instead of being a straight line passing through the origin, indicating that a trace amount (∼0.43% per mole of La_1.5_NiO_3.225_, calculated by comparing the 300 K isotherm step heights in Figure 3b and Figure S2) of ferromagnetic impurity with ∼0.43% molar ratio when compared to that of elemental Ni exists in the reduced sample La_3_Ni_2_O_6.45_, which is a known situation for topochemically reduced Ni-containing oxides.^20−22^ The subtly opened-up hysteresis loop in the 5 K isotherm further confirms the existence of ferromagnetic impurity in the system, which is equivalent to 0.43% by mole of elemental Ni (Figure 3b). Therefore, it is necessary to employ a ferro-subtraction technique, which can unveil the magnetic behavior of the bulk material by applying an external field greater than 2 T to saturate the magnetization of the ferromagnetic impurity. Thus, in 5 K increments between 5 and 300 K, field-dependent magnetization data were collected over the field range 3 T ≤ *H* ≤ 5 T and the resultant magnetizations (*M*) plotted as a function of the applied field (*H*). For each temperature, the slope of the plot obtained (i.e., ΔM/Δ*H*) by linear-fitting the data over the field range 3 T ≤ *H* ≤ 5 T can be considered as the paramagnetic susceptibility of the bulk material, since a saturation stage of ferromagnetic impurity starts to occur at field much smaller than 3 T (Figures S1 and S2**)**. Thus, a plot can be made by combining the data that were taken over the whole temperature range, which shows the temperature dependence of the bulk material’s susceptibility. In Figure 3c, the kink around 10 K may either be from the ferromagnetic impurity or a transition of some kind in the bulk material. The ferro-subtracted magnetic susceptibility curve shows one major phase transformation starting at *T* ≈ 50 K, which suggests that the bulk material has a relatively broad transition. A modified Curie–Weiss law (χ = *C*/(*T* – θ) + χ_0_) was used to fit the high-temperature region (100–250 K) of the inverse magnetic susceptibility data, to derive the Curie constant and the Curie–Weiss temperature of the bulk material (Figure 3d). The fitted Curie–Weiss temperature θ is 7.5(8) K, indicating the intrinsic weakly ferromagnetic average coupling in bulk La_3_Ni_2_O_6.45_. The calculated value of Curie constant C is 0.0705 cm^3^ K mol_Ni_^–1^ and yields an effective moment of 0.75 μ_B_ per Ni. The average oxidation state of Ni in the reduced phase is +1.95 according to the nominal composition, suggesting a combination of Ni^1+^ and Ni^2+^ in the localized picture. The theoretical effective moment per Ni is 1.73 μ_B_ for Ni^1+^ (*S* = 1/2) and 2.83 μ_B_ for Ni^2+^ (*S* = 1) based on the spin-only values for localized spins, which are significantly higher than the experimental value. The small magnitude of the experimental effective moment and the fact that the Curie–Weiss temperature is apparently lower than the magnetic ordering temperature, accompanied by the relatively broad shape of the observed transition in the ferro-subtracted magnetic susceptibility curve, indicate that future work on the magnetism of this material would be of interest.

**Figure 4:**
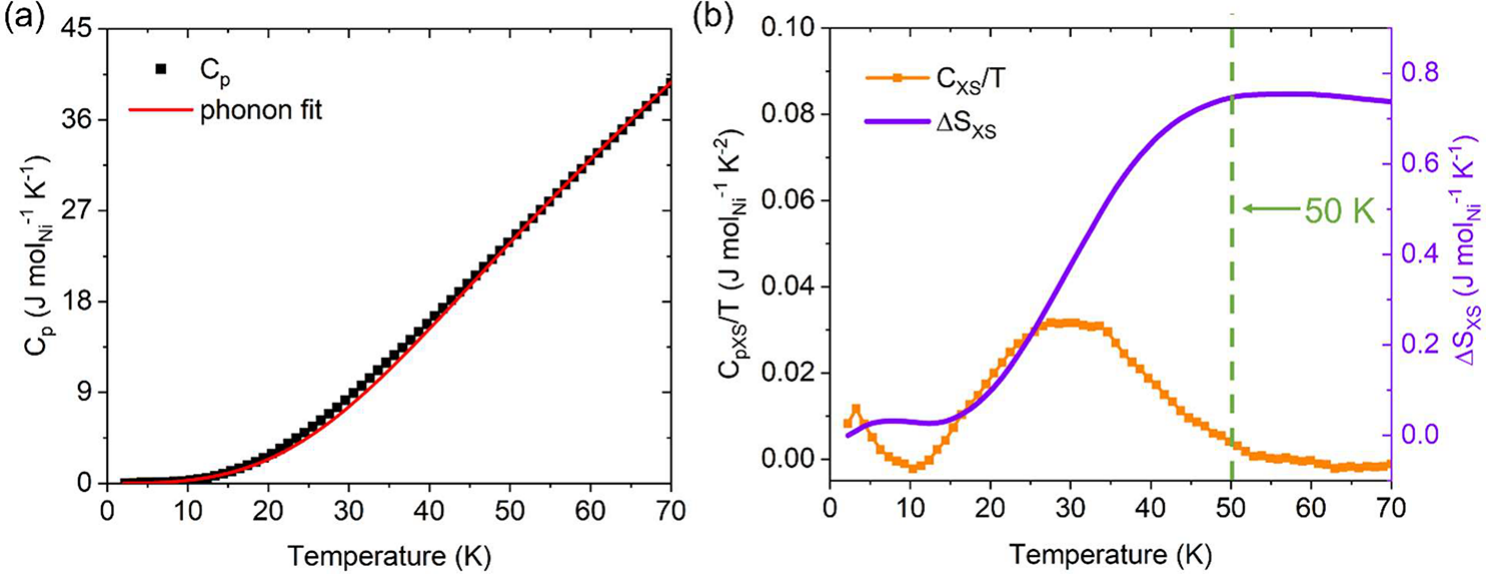
Heat capacity of La_3_Ni_2_O_6.45_. (a) Total heat capacity data *C*_p_ with the high temperature region fitted by a modified two-component Debye equation to estimate the phonon contribution. (b) Resulting *C*_mag_/*T* (orange) data, as well as the excess entropy change Δ*S*_XS_ (purple) plotted against temperature. The vertical dashed line at 50 K indicates that temperature.

Since we can still observe a broad hump in the magnetic susceptibility data after eliminating the contribution of a trace amount (∼0.43% per mole of La_1.5_NiO_3.225_) of ferromagnetic impurity, heat capacity data were then collected from a cold-pressed pellet of La_3_Ni_2_O_6.45_, to further investigate the nature of this anomaly. The collected heat capacity data were scaled to per-mole of Ni (based on the formula La_1.5_NiO_3.225_). The reduced phase La_3_Ni_2_O_6.45_ shows semiconducting behavior, consistent with reports in literature,^23^ and thus, there are few conduction electrons present that would yield a significant *C*_electron_. The total heat capacity data (*C*_total_) are plotted against temperature *T* in Figure 4a. There is no clearly defined peak in the heat capacity data, and therefore, the excess heat capacity collected under zero field in the temperature range of 50 to 70 K (above the magnetic transition), is estimated by fitting to a modified two-component Debye equation , in where *C*_1_ = 3.225, Θ_*D1*_ = 734(4) K, *C*_2_ = 2.5, and Θ_D2_ = 258(1) K, to estimate the phonon contribution, *C*_phonon_. The *C*_1_ and *C*_2_ values are in good agreement with the 2.5 heavy atoms (La and Ni) and 3.225 light atoms (O) in the formula La_1.5_NiO_3.225_. Note that the same two-component Debye equation has been applied to fit the high-temperature heat capacity of other oxide solids with coexisting light and heavy atoms.^24,25^ Therefore, after the phonon contribution *C*_phonon_ is subtracted from *C*_total_, the excess heat capacity contribution *C*_XS_ can be estimated. The resulting *C*_XS_/*T* (in orange) is plotted against temperature *T* in Figure 4b. The *C*_XS_/*T* curve shows a rise below 10 K, and a broad hump that starts at a temperature (50 K) similar to what we observed in the ferro-subtracted magnetic susceptibility data. Since magnetism is expected for a Ni^2+^-based system, we can reasonably attribute the heat capacity excess to a magnetic transition and as such the magnetic entropy change Δ*S*_xs_ (in purple) can be calculated by taking the integral of *C*_XS_/*T* over the measured temperature range, yielding a saturation value of ∼0.75 J/mol_Ni_/K, which is small even when compared to the Ising spin prediction *R* ln (2) = 5.76 J/mol/K. Whereas the heat capacity data does suggest that the anomaly seen in the temperature-dependent magnetization data are magnetic in origin, more work is needed to determine nature of the apparent magnetic ordering at low temperature if present.

**Figure 5:**
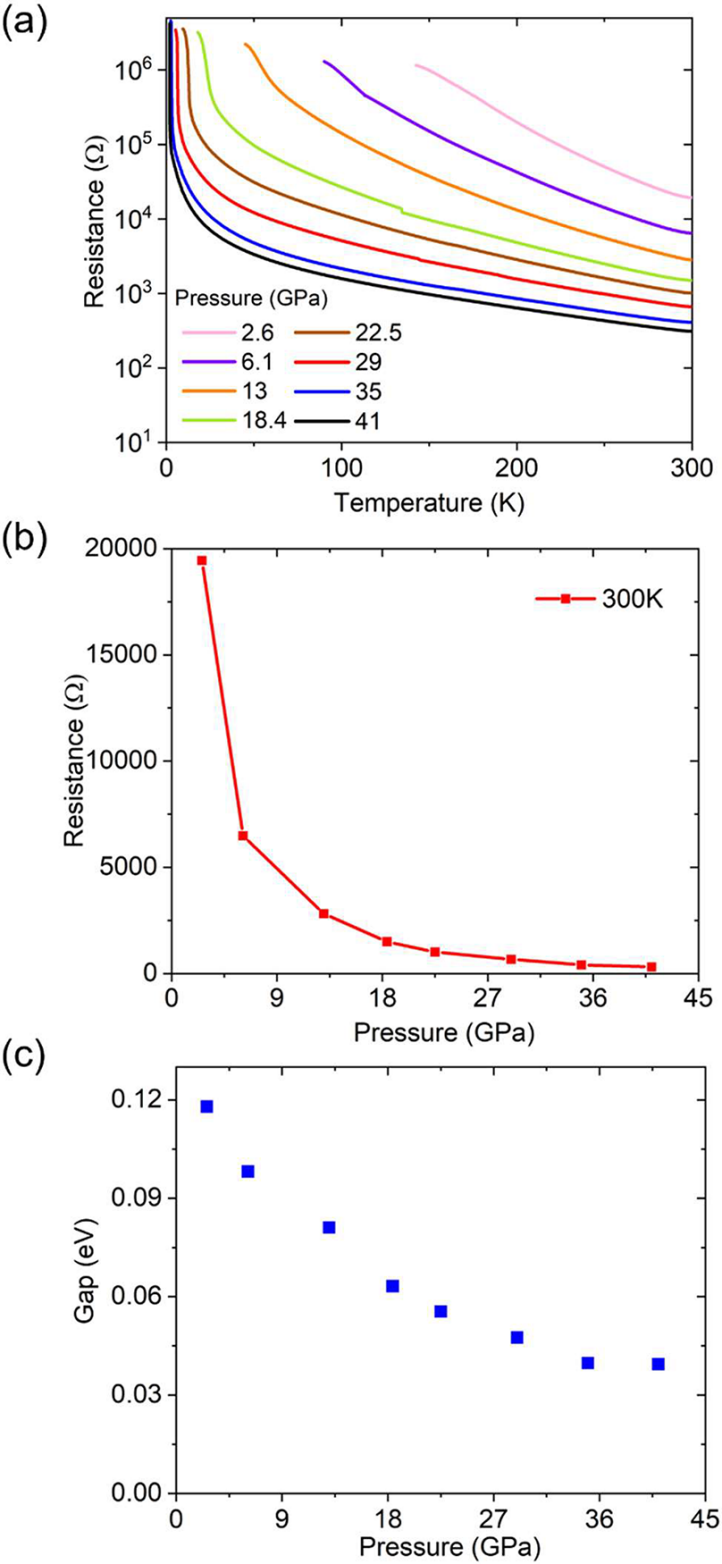
Pressurized resistance of La_3_Ni_2_O_6.45_. (a) Temperature-dependent resistivity at various applied pressures. (b) Ambient temperature resistance plotted against applied pressure. (c) Resistively determined energy gap as a function of pressure.

The reduced phase La_3_Ni_2_O_6.45_ prepared in the present work is a semiconductor at ambient pressure, consistent with previous descriptions.^23,26^ However, given the fact that La_3_Ni_2_O_6.93_ was recently reported to show signatures of superconductivity under high applied pressure,^12^ the temperature-dependent resistivity of La_3_Ni_2_O_6.45_ under pressure is of interest as well. Unfortunately, no signs of superconductivity have been detected for our La_3_Ni_2_O_6.45_ under applied pressures of up to 41 GPa. The material remains a semiconductor when pressurized, which we deduce from the fact that the material shows an increase in resistance on a decrease in the temperature under all pressures studied (Figure 5a). The resistance at ambient temperature is found to drop significantly under applied pressure, with the largest changes occurring below 9 GPa (Figure 5b). Also, the semiconducting gap (Figure 5c) obtained from the fits of the high temperature part of resistance curves (Figure S2) decreases under pressure by a factor of ∼3, suggesting that applied pressure brings La_3_Ni_2_O_6.45_ significantly closer to a metallic state than it is at ambient pressure.

## Conclusion

In the present work, reduced phase La_3_Ni_2_O_6.45_ was synthesized from the parent La_3_Ni_2_O_7_ phase by heating in TGA under different reduction conditions. The reduction process was studied through a combination of PXRD and TGA data, offering insights into the structural changes between La_3_Ni_2_O_7_ and La_3_Ni_2_O_6.45_. The intrinsic magnetic properties of bulk La_3_Ni_2_O_6.45_ were unveiled by saturating the inevitably present ferromagnetic impurity under a high external applied field using a so-called ferro-subtraction technique. The broad hump observed in the ferro-subtracted magnetic susceptibility curve starting at a temperature around 50 K, accompanied by the broad anomaly at a similar temperature in the *C*_XS_/*T* curve, confirms the intrinsic behavior of the reduced material. No sign of superconductivity was found in our reduced phase La_3_Ni_2_O_6.45_, although for superconductors, it has been demonstrated that signs of superconductivity can be observed even when the composition of the superconductor is not the composition of the sample due to the inevitable composition variations present in real materials. Instead, we introduce an intrinsic transition to the otherwise weakly paramagnetic La_3_Ni_2_O_7_ and shed light on the reduction profile of the parent oxide compound, which is currently in the spotlight due to recent reports of it displaying high pressure superconductivity. Our work shows that the scientific community should consider whether both the amount and arrangement of oxygen vacancies may be crucial for the nickelates to hold superconductivity, Thus, our study encourages the more detailed study of formulas with different oxygen contents in both nickelates and other oxides that are challenging to stably obtain.

## Supporting Information Available

High-field fitting of magnetization (*M*) against field (*H*) data for ferro-subtraction. Field-dependent magnetization data of pure Ni powder at 300 K. Resistance of reduced phase La_3_Ni_2_O_6.45_ plotted against 1/*T* under different pressures. (PDF)
