# Supplementary material for: Is La3Ni2O6.5 a Bulk Superconducting Nickelate?
Source: ACS Appl Mater Interfaces. 2024 Feb 21;16(49):66857–64. doi: 10.1021/acsami.3c17376 (PMC11647759; doi:10.1021/acsami.3c17376)
Supplement: Supplementary file 1 — am3c17376_si_001.pdf [file am3c17376_si_001.pdf]

Supporting Information for

Is $La_3Ni_2O_{6.5}$ a Bulk Superconducting Nickelate?

Ran Gao[1#], Lun Jin[1#], Shuyuan Huyan[2,3#], Danrui Ni[1*], Haozhe Wang[4], Xianghan Xu[1], Sergey L. Bud'ko[2,3], Paul Canfield[2,3], Weiwei Xie[4*] and Robert J. Cava[1*]

[1]Department of Chemistry, Princeton University, Princeton, New Jersey 08544, USA

[2]Ames National Laboratory, Iowa State University, Ames, IA 50011, USA

[3]Department of Physics and Astronomy, Iowa State University, Ames, IA 50011, USA

[4]Department of Chemistry, Michigan State University, East Lansing, Michigan 48824, USA

* E-mails of corresponding authors: xieweiwe@msu.edu; danruin@princeton.edu; rcava@princeton.edu

# L.J., R.G. and S.H. contributed equally.

Table of Contents

Figure S1. High-field fitting of magnetization (*M*) against field (*H*) data for ferro-subtraction. Note that all panels are plotted on the scale "per mole Ni", not "per mole formula unit" ……..... **S2**

Figure S2. Field dependent magnetization data of pure Ni powder at 300 K. Note that the data are plotted on the scale "per mole Ni", not "per mole formula unit" ……………………………….. **S3**

Figure S3. Resistance of the reduced phase $La_3Ni_2O_{6.45}$ plotted against 1/T under different pressures…………………………………………………………………………………….**S4**

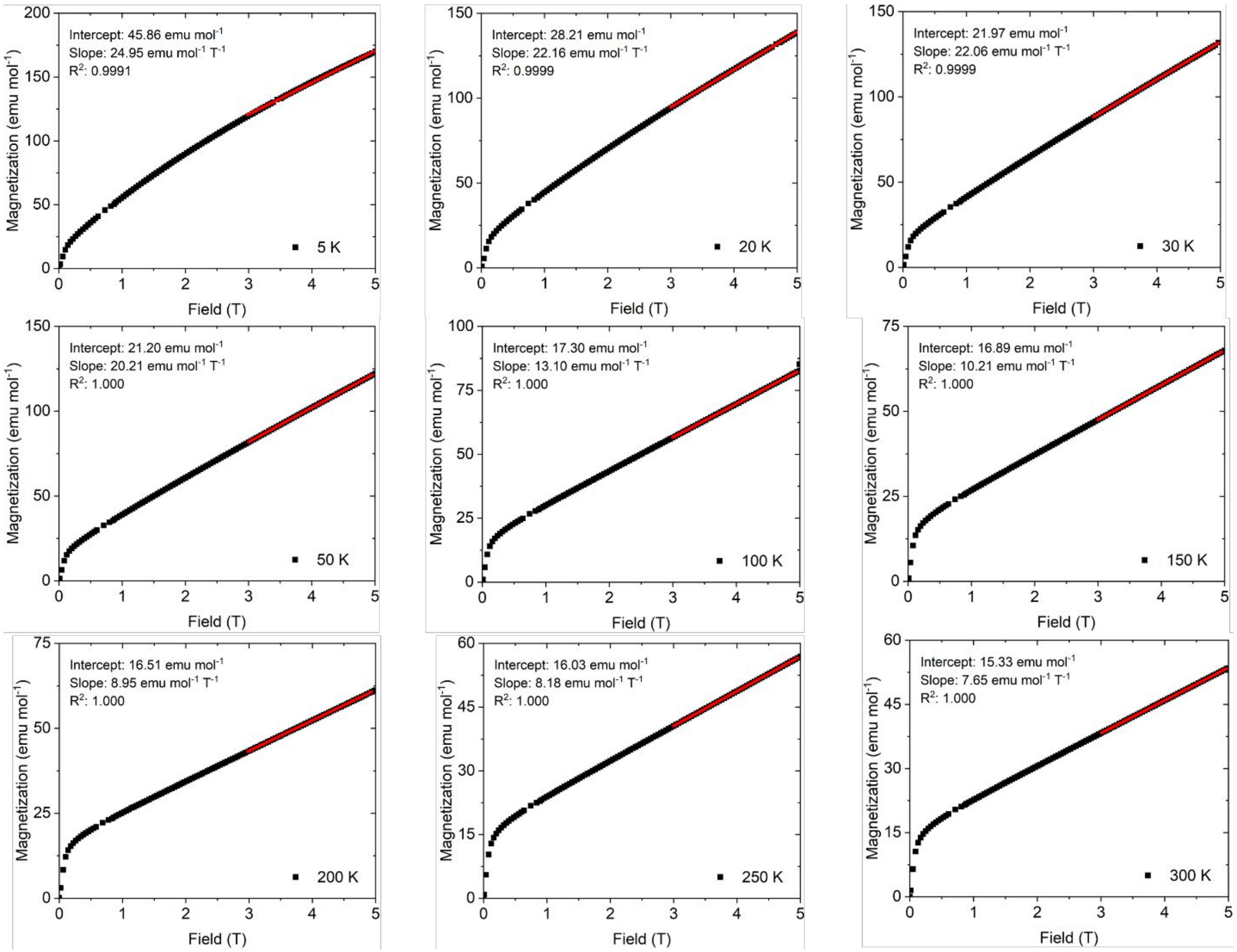

Figure S1. High-field fitting of magnetization (*M*) against field (*H*) data for ferro-subtraction. Note that all panels are plotted on the scale "per mole Ni", not "per mole formula unit".

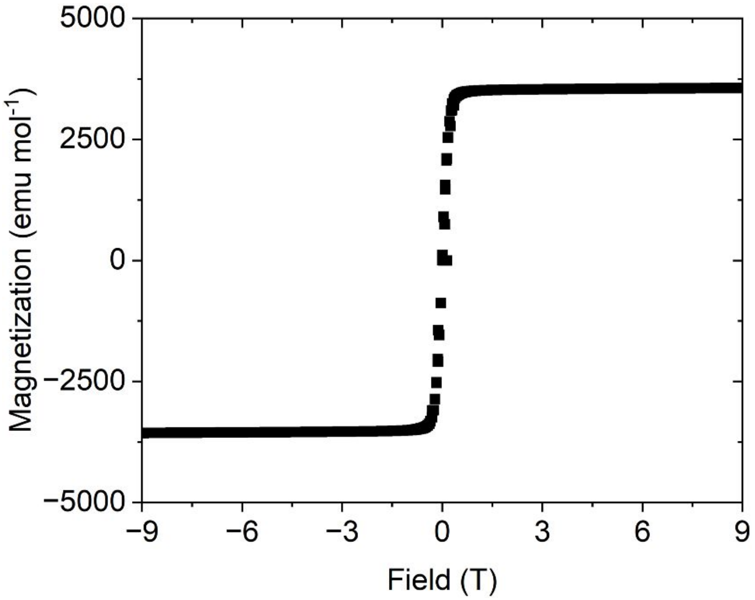

Figure S2. Field dependent magnetization data of pure Ni powder at 300 K. Note that the data are plotted on the scale “per mole Ni”, not “per mole formula unit”.

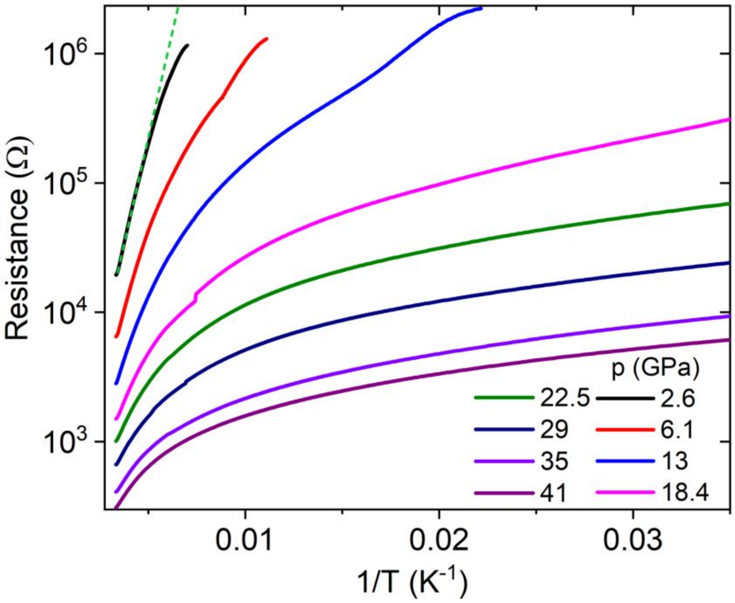

Figure S3. Resistance of the reduced phase $La_3Ni_2O_{6.45}$ plotted against 1/T under different pressures.
